# The Self-Sealing Capacity of Environmentally Friendly, Highly Damped, Fibre-Reinforced Concrete

**DOI:** 10.3390/ma13020298

**Published:** 2020-01-09

**Authors:** Xu Huang, Jun Ge, Sakdirat Kaewunruen, Qian Su

**Affiliations:** 1 Department of Civil Engineering, School of Engineering, University of Birmingham, Birmingham B152TT, UK; XXH689@student.bham.ac.uk; 2 Laboratory for Track Engineering and Operations for Future Uncertainties (TOFU Lab), School of Engineering, University of Birmingham, Birmingham B152TT, UK; JXG826@student.bham.ac.uk; 3 School of Civil Engineering, Southwest Jiaotong University, Chengdu 610031, China; suqian@126.com

**Keywords:** self-sealing concrete, ultrasonic pulse velocity, fibre-reinforced concrete, environmentally friendly materials, highly damped concrete, rubberized concrete

## Abstract

Cracks could attenuate the service life of concrete structures because of the intrusion of hazardous substances such as water. In this study, different proportions of Duras S500 fibre were employed to investigate the self-sealing capacity of environmentally friendly, highly damped, fibre-reinforced concrete (EFHDFRC) containing 5% crumb rubber. The workability of EFHDFRC with different proportions of the fibre was investigated by mechanical properties test. The self-sealing capacity was first measured by introducing the ultrasonic pulse velocity (UPV) test combined with the damage degree in a time-dependent manner. In addition, the regained compressive strength test and visual inspection were applied as additional measures of the self-sealing capacity. The experimental results show that EFHDFRC with different proportions of fibre showed the maximum sealing degree between the 42nd and 51st days after casting the concrete. EFHDFRC with 0.1% fibre had the best performance and the maximum self-sealing degree (2.82%). In summary, it has been proven that 0.1% fibre could stimulate the self-sealing capacity of EFHDFRC by bridging cracked concrete. Moreover, it is noted that sufficient space in cracks is essential for precipitation formation, which could seal the cracks. The new insights of this innovative self-healing, high-damping material are essential for industrial applications exposed to dynamic load conditions such as railway turnout bearers and sleepers, highspeed rail track slabs, blast-resistant walls and columns, and so on.

## 1. Introduction

Concrete is a popular material worldwide because of its high compressive strength, low cost, and high stiffness [1]. However, the durability of concrete could be negatively affected by cracks, which can easily be caused by impact loading, shrinkage, and detrimental environmental exposure [2]. Carbon dioxide, water, and chloridoid could permeate concrete to cause corrosion and carbonation through cracks [3]. It is evident that the presence of cracks not only leads to a certain degree of degradation of penetration resistance against chloride and water, but also shortens the service life and gives rise to a loss of stiffness in concrete structures [4]. The conventional way to repair cracks in concrete structures is to artificially inject aided repairing materials such as epoxy resin. However, there are some drawbacks to this method. Firstly, although these repairing materials could provide excellent performance in terms of strength recovery, their negative influence on the environment cannot be ignored. To some extent, most repairing materials are toxic. For instance, polyurethane material could produce a significant amount of substances such as phenol and chlorofluorocarbons that are not environmentally friendly [5]. Secondly, it is difficult to inspect and repair cracks in places where access is difficult, such as concrete sleepers. Thirdly, the maintenance fees are costly. In the UK, half of a construction fee is devoted to the maintenance of existing concrete structures to prolong their service life [6]. Moreover, it is pointed out that the cost of concrete ranges from €60 to €75 per m^3^, which is relatively cheap. However, the maintenance fees for cracked concrete structures could be over €130 (direct costs) per m^3^ [7]. Thus, self-repairing concrete was proposed to reduce the effect of cracks and enhance the durability of concrete structures.

**Figure 1:**
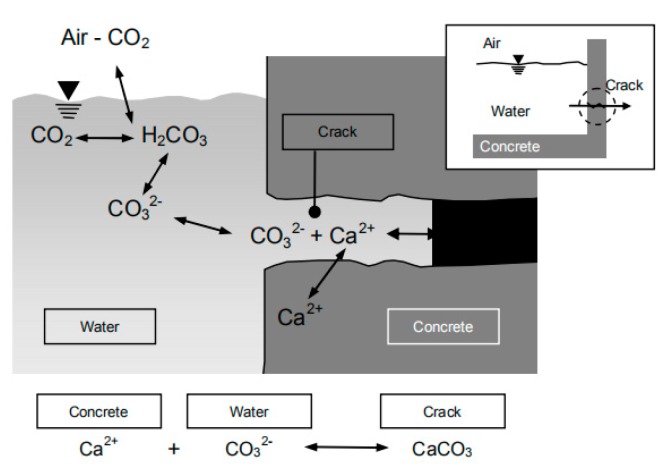
Self-repairing mechanism (ongoing hydration process) [9].

Self-repairing concrete has been a subject of research attention since it was first presented by Dry in 1994 [8]. The self-repairing mechanisms can be described as follows. Firstly, with regard to young concrete, unreacted cement particles can hydrate to form calcium carbonation precipitation, as shown in Figure 1 [9]. For older concrete, carbon dioxide obtained from the cement hydration can react with calcium hydroxide to produce calcium carbonation precipitation. Secondly, debris in water could seal cracks [10]. Self-repairing can be divided into two methods, self-healing and self-sealing. The difference between self-healing and self-sealing is whether the mechanical properties recover. The self-healing method was classified as autogenous healing and autonomic healing [11]. Beyond that, it is divided into three aspects: intrinsic healing, vascular healing, and capsule-based healing [8]. The autogenous healing method relies on chemical or physical substances stored in concrete to seal cracks, which is the same as intrinsic healing. By comparison, vascular healing and capsule-based healing deliver healing agents to repair cracks such as epoxy resin, acrylics, polyurethanes and *Bacillus sphaericus* LMG 22557 by employing hollow tubes and capsules, respectively [5,12,13,14]. To simplify the classification, self-healing concrete is referred to as either autogenous healing or agent-based healing in this article. According to previous research, the agent-based healing method can heal cracks smaller than 970 μm, while cracks no wider than 320 μm could be sealed by the autogenous healing method [15]. However, most of the chemical healing agents utilised for agent-based healing are environmentally unfriendly, as mentioned before. Moreover, maintaining plastic tubes and capsules during the service life of concrete structures is also problematic [16,17]. Thus, the self-sealing method inspired by autogenous healing is studied in this article.

With regards to previous studies on autogenous sealing, magnesium and calcium were explored to test their influence on the self-sealing capacity [18]. Moreover, a shape memory alloy (SMA) was found able to help seal cracks with a 14.3% and a 15.8% crack width reduction after heating at 56 °C and 81 °C, respectively [19]. Meanwhile, fibre-reinforced concrete was investigated as to the feasibility of sealing cracks. Homma noted that the combination of steel fibre and polyethene conferred the highest self-sealing ability [20]. Nishiwaki et al. mentioned that 1.5% polyethene and polyvinyl alcohol showed the same self-sealing ability when the crack width was smaller than 100 μm. However, concrete with polyvinyl alcohol had a better self-sealing capacity when the crack width was over 100 μm [21]. In summary, fibre could strengthen the self-sealing capacity of concrete for small cracks. In addition, since concrete is relatively brittle and its poor performance under occasional impact loading is acknowledged, it is necessary to improve the damping capacity of concrete to sufficiently abate the effect of impact loading. Kaewunruen et al. mentioned that 5% of (180 and 400 μm) crumb rubber with 10% silica fume showed the best mechanical properties and damping capacity [22]. Furthermore, rubber, whose main component, styrene, affects the eyes and the upper respiratory tract when it is burned, is estimated to be the second-largest waste material in the world. If not reused, waste rubber would cover a significant amount of land [23]. Duras S500 fibre incorporating 5% crumb rubber was studied in this article to explore the self-sealing capacity of environmentally friendly, highly damped, fibre-reinforced concrete (EFHDFRC). The expected self-sealing mechanism in this article is to bridge cracked samples by employing additional fibre. The principle of the mechanism is that fibre could confine cracked samples when cracks are generated and then the possibility of crack expansion can be reduced. Therefore, the chance to form precipitation that can fill in cracks to stimulate the self-sealing capacity can be enhanced.

As regards the self-sealing capacity, four major aspects, the crack size, the sealing degree, the durability test, and the mechanical properties recovery, were taken into consideration. There have been several self-sealing capacity measurements reported. For instance, microscopes and scanning electron microscopes (SEM) were employed to monitor the crack width [24,25]. There is only one scholar who has researched the crack depth [15]. However, it is not sufficient to evaluate the self-sealing capacity by inspecting either the crack width or the crack depth. The reason is that cracks do not propagate linearly, but contain different widths and depths. Therefore, it is tough to precisely measure changes in the volume of cracks, the volume of initial cracks, and the percentage of sealed concrete. Furthermore, water permeability and gas permeability were used to calculate the self-sealing capacity. However, the changes in weight in these methods are tiny (e.g., 0.1 g) and difficult to measure accurately [26].

In order to improve the accuracy of self-sealing capacity evaluation, an ultrasonic pulse velocity (UPV) instrument (PUNDIT 200, supplied by Proceq UK Limited, Bedford, UK) was used in this study. A UPV instrument is usually applied to inspect the integrity of concrete. Researchers have proven that there is a connection between damage in concrete and a decline in UPV results [27,28]. The principle behind this is that the ultrasound would perform at different speeds when traveling through different substances, such as water, concrete, and air. Thus, the ultrasound velocity variation would reflect the inner changes of concrete. Zhong and Yao first employed a UPV instrument testing on the damage degree to inspect initial cracks and the self-healing ratio of concrete, as defined by applying the regained compressive strength method. A threshold was found in that the self-healing ratio increased in parallel with the damage degree up to a certain point [26]. In this article, a UPV instrument was first employed to test both the damage degree and the self-sealing degree of concrete.

The purpose of this article is to investigate the influence of added fibre contents on the self-sealing capacity of environmentally friendly, highly damped, fibre-reinforced concrete. The development of the novel concrete is necessary for concrete structures to produce self-sealing environmentally friendly, highly damped concrete, which could automatically seal small cracks to considerably enhance the durability of concrete structures, thus reducing maintenance costs and effectively attenuating the effects of impact loading. Five percent crumb rubber (180- and 400-micro), recommended by Kaewunruen et al. [22], was added to produce eco-friendly, highly damped concrete. Different proportions of the fibre Duras S500 were applied to analyse their influence on the self-sealing capacity. Unlike previous scholars who measured the self-sealing capacity by applying crack width evaluation and durability evaluation, the damage degree and the time-dependent self-sealing degree obtained by the UPV instrument were combined in this article to accurately and continuously measure the self-sealing capacity. Moreover, the regained compressive strength method and visual inspection were employed as additional measures to assess the self-sealing capacity.

## 2. Experimental

### 2.1. Materials

**Figure 2:**
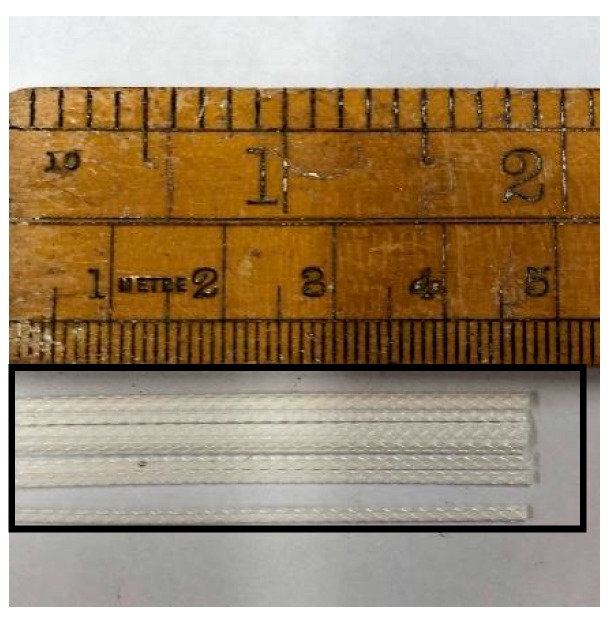
Duras S500 fibre.

**Figure 3:**
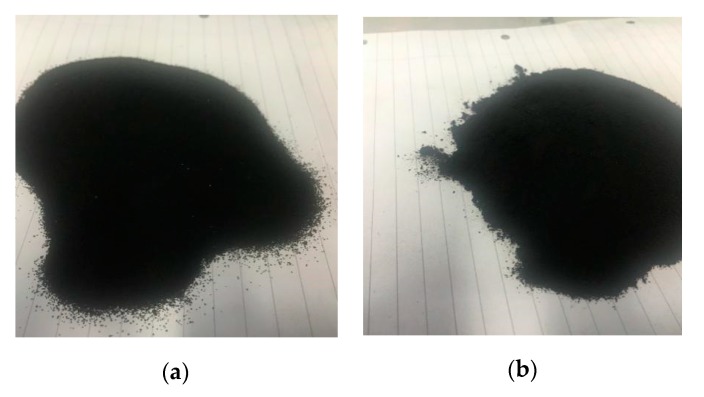
Crumb rubber (**a**) 180-micron; (**b**) 400-micron.

The construction fibre shown in Figure 2, DURUS S500, was provided by the ADFIL Construction Fibre Company, Zele, Belgium. The properties of the fibre are listed in Table 1. Moreover, Ordinary Portland Cement type I (CEM I 52.5 N) was employed as a binder for casting concrete samples in accordance with BS EN 197-1 [29]. Furthermore, aggregates smaller than 4.75 mm were applied as fine aggregates according to BS EN 12620:2002 [30]. Moreover, tap water from the Civil Engineering Laboratory of the University of Birmingham (Birmingham, UK) was used as the batch water in this article. The 180- and 400-micron crumb rubbers provided by Lehigh Technologies Incorporation (Atlanta, GA, USA) were mixed at a ratio of 1:1, as shown in Figure 3, to substitute for 5% of the sand.

### 2.2. Concrete Sample Preparation and Crack Generation

This section gives guidance on how to prepare EFHDFRC samples and generate residual cracks. Table 2 illustrates the design of concrete, which follows the work of Kaewunruen et al. The water‒cement ratio selected was 0.44. Moreover, the slump values were recorded around 80 ± 20 mm. Mix 1 was defined as the reference concrete batch without the fibre and crumb rubber; 5% of crumb rubber was added into the concrete samples of Mix 2. From Mix 3 to Mix 7, 5% crumb rubber and fibre ranging from 0.1% to 0.4% replaced the mass of sand and coarse aggregates. The gradation of aggregates is demonstrated in Table 3. The testing details of each experiment in this article are given in Table 4.

**Figure 4:**
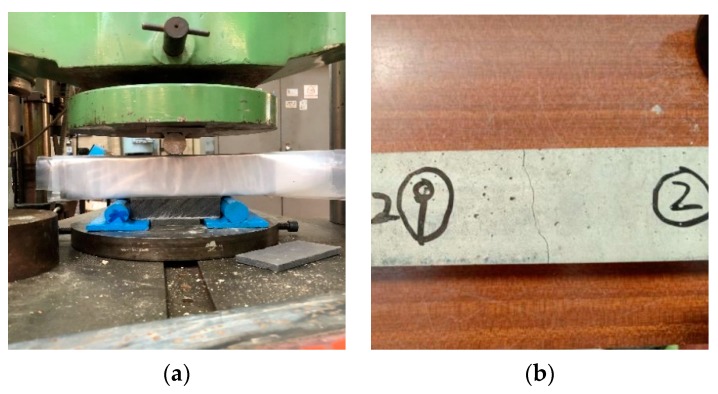
(**a**) MTS; (**b**) realised cracks.

To obtain the self-sealing capacity results, precracking of samples (50 × 50 × 250) is essential. The reasons why precracking is required are as follows. Firstly, the bridging function to stimulate the self-sealing capability of the fibre can be investigated only if there are cracks in concrete samples. If there is no crack in the concrete samples, no changes in crack size can be detected to identify the self-sealing capacity. Secondly, realised cracks could act as a simulation of the cracks that appear in concrete structures during daily operation. Thus, the self-sealing capacity of the novel concrete can be precisely measured by employing realised cracks. With regard to the self-sealing capacity evaluation, the processes of concrete samples’ preparation and the crack generation method are as follows. Firstly, fresh samples (50 mm × 50 mm × 250 mm) were placed in the laboratory at room temperature (25 °C) for 24 h. Secondly, they were demoulded and put into a curing tank filled up with tap water for 28 days. On the 28th day, the samples were mounted on the MTS instrument (MN, USA) shown in Figure 4a for crack generation. Several scholars employed a three-point or four-point bending machine for crack simulation [31,32]. However, no detailed process of crack generation has been reported. According to preliminary tests done by the author, concrete samples were easily crushed into several pieces by loading, especially for non-reinforced concrete. Therefore, the novel method to generate realised cracks is as follows. Firstly, a pilot specimen was primarily tested by the MTS with a loading rate at 0.02 Mpa/s in order to record the yield flexural strength. Next, the loading pad of the machine was dropped smoothly to other samples of the same mix with the same setup of the MTS before the yield flexural strength. Finally, the loading pad was kept steady at 1 Mpa less than the yield flexural strength and visual inspection was employed to observe the crack appearance. Cracks in the No. 9 sample of Mix 2 are shown in Figure 4b. The initial crack widths of concrete in each mix are given in Table 5.

## 3. Tests and Methods

### 3.1. Mechanical Properties

#### 3.1.1. Compressive Strength

**Figure 5:**
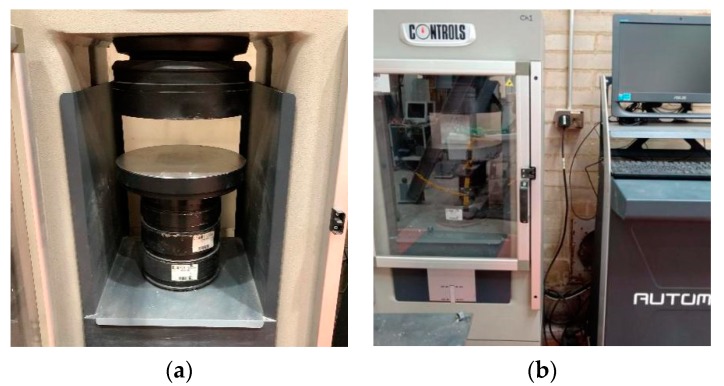
(**a**) Compressive test setup; (**b**) Controls Automax machine for the compressive strength tests.

The compressive strength test setup is shown in Figure 5a. The test was conducted by applying a machine called Controls Automax (Milan, Italy) (shown in Figure 5b) based on BS EN 12390-3 [33]. Nine cubes (100 mm) of the same mix were tested as a batch on the 7th, 14th, and 28th days. In order to obtain more accurate compressive strength measurements, the cubic samples were heated in an oven at 80 °C for 1 h and then cooled to room temperature before testing. Furthermore, both surfaces of the loading pad and cubes were cleaned to avoid errors caused by eccentric compression.

#### 3.1.2. Splitting Tensile Strength

**Figure 6:**
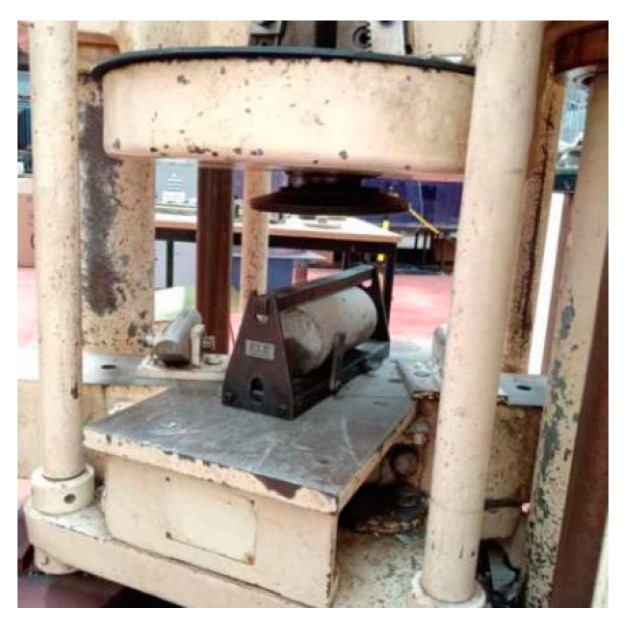
Splitting tensile test setup.

Based on BS EN 12390-6 [34], three cylindrical samples (Ø100 mm × L 200 mm) were utilised to measure the splitting tensile strength of concrete on the 28th day [34]. The machine for testing the splitting tensile strength was called Avery-Dension, AD-T42 (OH, USA), shown in Figure 6. In order to implement the test, samples were heated and cooled and then the surfaces of the samples and the machine were cleaned. Moreover, the press bar of the machine should be placed in the middle of the loading pad and the samples. The descending rate of the loading pad was kept steady and uniform at 0.02 to 0.05 MPa/s until the samples failed and then the splitting tensile strength value was recorded.

#### 3.1.3. Flexural Strength

Three prisms (W100 mm × H100 mm × L500 mm) were tested on the 28th day after casting concrete by employing the same machine for testing the splitting tensile strength with the same loading rate, in accordance with BS EN 12390-5 [35]. The difference was that the loading pad for the splitting tensile strength test was changed to two loading rollers.

### 3.2. Self-Sealing Capacity

#### 3.2.1. Crack Size Measurement

1. Visual Inspection

The self-sealing capacity of EFHDFRC was investigated through the visual inspection method, employing a film card. The principle of this method is to roughly investigate the changes in crack width on the surface of concrete samples. Crack widths in fibre-reinforced concrete samples, from M2 to M7, were measured on days 35, 42, and 56 after casting concrete in this article. The film card was placed near cracks and the closest width value was selected as the crack width. Subsequently, a camera was employed to record the widths of cracks.

2. Damage Degree

**Figure 7:**
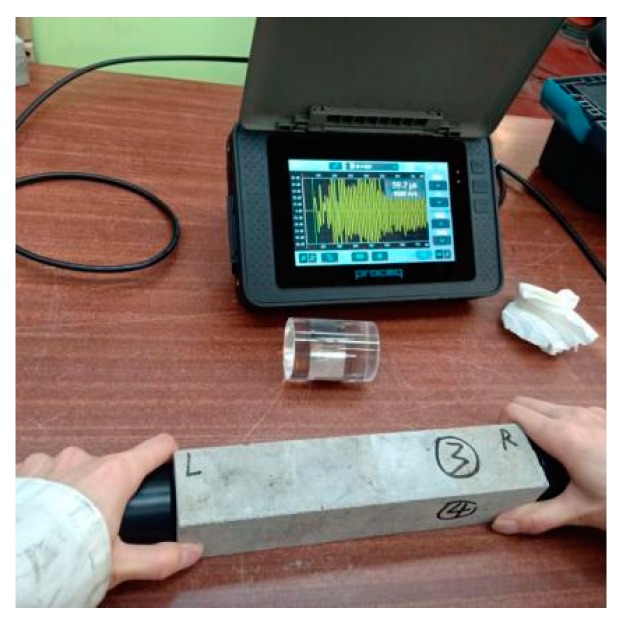
Proceq Pundit 200 ultrasound pulse velocity instrument.

The study of Suaris and Fernado demonstrates that the value of UPV testing decreases with continuous compressive loading [36]. The concept of the damage degree to indicate initial crack sizes was first proposed by Zhong and Yao. The paper mentions that there are damage degree thresholds of high-strength and normal-strength concrete. The thresholds are 0.4 and 0.6–0.7 for high strength and normal strength concrete, respectively. One possible reason why the threshold of normal-strength concrete is higher than that of high-strength concrete is that the strength increment of normal-strength concrete is less than that of high-strength concrete. Thus, more unreacted cement particles remained when cracks are generated. Moreover, when the damage degree is lower than the threshold, the self-sealing ratio increases with the rising damage degree. The self-sealing ratio decreases with the increase of the damage degree when the damage degree is higher than the threshold. One possible explanation may be that when the damage degree is small, unreacted cement particles cannot be exposed to enough rehydration to seal the cracks. However, when the damage degree exceeds the threshold, rehydration products cannot fill in the cracks [26]. However, the UPV method was not used to test the self-sealing ratio directly. Importantly, the influence of time on the compressive strength of concrete was not taken into account when testing the self-sealing ratio. Furthermore, there was not enough data to describe the trend of the self-sealing ratio because the scholar only applied four times the compressive strength measurement to define the self-sealing ratio within 60 days. This means that the method might not show an accurate, time-dependent self-sealing ratio of concrete samples. In this article, the damage degree and the time-dependent self-sealing degree were measured to investigate the relationship between them by employing a UPV instrument named Pundit 200 from Proceq (Schwezenbach, Switzerland) as shown in Figure 7 before and after crack generation. Then, the damage degree was defined according to Equation (1).

(1) $$D=\left(V_{0}-V\right)/V_{0},$$

where D = Damage degree, $V_{0}$ = the ultrasound velocity before cracking, and V = the ultrasound velocity after cracking.

#### 3.2.2. Regained Compressive Strength

Based on previous studies, regained mechanical properties were applied to identify the self-sealing capacity and the self-healing capacity over a long time. A few scholars had employed the regained flexural strength as the indication of the self-repairing phenomenon [37]. Most of them had chosen the regained compressive strength test [26,38]. However, the major issue with the regained compressive strength measurement is that most scholars only tested the compressive strength on the 28th day after crack generation. This means that only the percentage of the regained compressive strength on the 28th day could be reflected. According to the self-sealing capacity research, most of the cracks were sealed within 14 days. Thus, it is meaningful to test the regained compressive strength in the first 14 days after the crack appearance. In this article, three concrete cubes as were tested reference samples for the deviation reduction on the 28th, 35th, and 42nd days after crack generation in accordance with BS EN 12390-3 [33]. The other six concrete cubes (cracked samples) were implemented with the compressive strength test on the day of the crack appearance and then three of them were tested separately for the regained compressive strength on the 35th and 42nd days.

The difference in compressive strength between reference and cracked samples on the 35th and 42nd days was evaluated by employing Equation (2). The principle of the regained compressive strength test is shown in Table 6.

(2) $$RCS=\left[\frac{C-G}{C}-\frac{A-F}{A}\right]-\left[\frac{B-E}{B}-\frac{A-D}{A}\right],$$

where A = the average compressive strength of reference samples on day 28, B = the average compressive strength of reference samples on day 35, C = the average compressive strength of reference samples on day 42, D = the average compressive strength of samples A, B, and C on day 28, E = the average compressive strength of samples A, B, and C on day 35, F = the average compressive strength of samples D, E, and F on day 28, and G = the average compressive strength of samples D, E, and F on day 42.

#### 3.2.3. Time-Dependent Self-Sealing Degree

A UPV instrument was first applied to directly test the time-dependent self-sealing degree combined with the damage degree. The principle for measuring the self-sealing degree with the UPV instrument is to compare the ultrasonic velocities passing through concrete and cracks. To obtain more precise data, steps for testing the self-sealing degree needed to be followed. Firstly, pilot samples were heated at 80 °C for 1 h and then cooled to room temperature before conducting the UPV test in order to decrease the influence on the UPV value of different moisture contents and temperatures. Guneyli et al. mentioned that 1% extra water could result in 160 m/s growth on the UPV value. Moreover, the UPV results increased 34 m/s with a temperature rise of 10 °C [39]. Secondly, the UPV equipment was calibrated before and during the testing procedure. Thirdly, testing of the same prism was conducted four times with different directions to obtain more accurate UPV values. Finally, the UPV test was applied to the same prism every three days for 28 days after concrete casting. Equation (3) was employed to convert the UPV value from ultrasound transferring time to ultrasonic velocities according to BS EN 12504-4:2004 [40]:

(3) V=LT,

where V = ultrasound velocities, L = the length of samples, and T = Transferring time.

As mentioned in the introduction, the UPV instrument has not been applied to directly test the self-sealing capacity. An equation to transfer the UPV value to the self-sealing capacity of concrete is presented in Equation (4). Firstly, the UPV values of four samples were measured in the same mix. One of the samples was recognised as the reference one. Secondly, the difference in the ultrasound values between reference and cracked samples in the same mix was calculated. Then, by comparing the difference in the UPV values of reference and cracked samples between the crack appearance on the first day and *n* days after sealing, the time-dependent self-sealing degree can be calculated.

(4) TDSSD=(Vnr−Vnc)Vnr−(V0r−V0c)V0r,

where TDSSD = the time-dependent self-sealing degree, $V_{nr}$ = the ultrasound velocities of reference samples on day *n*, $V_{nc}$ = the ultrasound velocities of cracked samples on day *n*, $V_{0r}$ = the ultrasound velocities of reference samples on the crack appearance day, and $V_{0c}$ = the ultrasound velocities of cracked samples on the crack appearance day.

## 4. Results and Discussion

### 4.1. Mechanical Properties

#### 4.1.1. Compressive Strength

**Figure 8:**
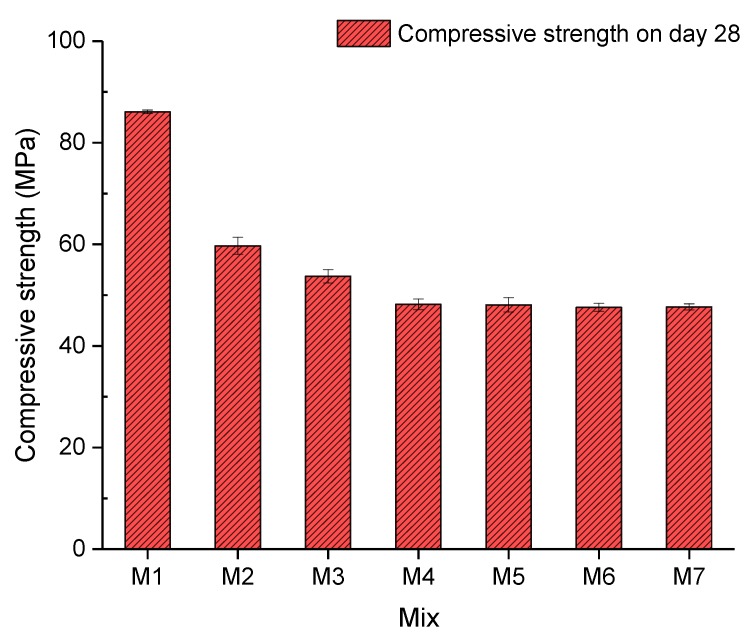
Compressive strength.

The compressive strength of concrete on the 28th day is shown in Figure 8. Research indicates that rubber could negatively affect the compressive strength of concrete [41,42]. A significant decrease (30.66%) in the compressive strength between Mix 1 and Mix 2 with 0% and 5% crumb rubber can be noticed in Figure 8. Subsequently, the compressive strength of Mix 3 and Mix 4 decreased at rates of 10.02% and 10.32%, respectively. It can be concluded that the downward trend of the compressive strength caused by adding 5% crumb rubber decelerated when 0.1% and 0.2% fibre was added. A possible reason for this deceleration could be that the fibre could strengthen concrete specimens to make the tested concrete bear higher loads than plain concrete. Afterwards, by incorporating 0.2%, 0.3%, 0.35%, and 0.4% of crumb rubber, the compressive strength of concrete from Mix 4 to Mix 7 fluctuated around 48 MPa, which can be interpreted to mean that over 0.2% of fibre cannot improve the compressive strength. Less than 0.1% fibre could improve the compressive strength by means of bridging cracked concrete. However, when gravels, which can take comparatively higher loads, were excessively substituted by fibre, the overall compressive strength of concrete was weakened.

#### 4.1.2. Splitting Tensile and Flexural Strength

**Figure 9:**
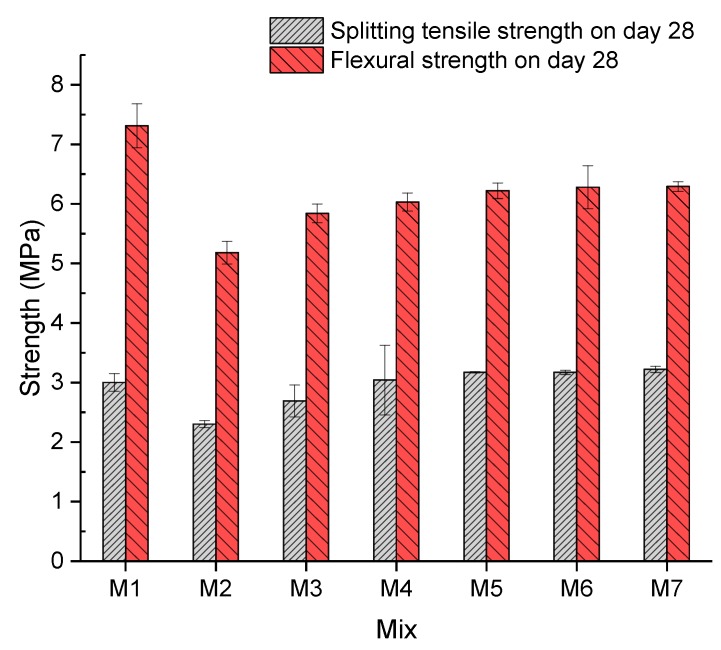
Splitting tensile and flexural strength.

In accordance with Figure 9, a similar trend could be observed between the splitting tensile and flexural strength of the concrete. An observable downward trend (23.25% and 29.20%) was caused by adding 5% crumb rubber of both strengths between Mix 1 and Mix 2, as shown in Figure 9. It can be concluded that additional crumb rubber can negatively influence mechanical properties. A possible explanation could be that a reduced amount of sand results in a decrease in strength because sand is the skeleton of concrete and so is significant to the compactness of concrete. Moreover, sand can fill the gaps between coarse aggregates to increase mechanical properties. Subsequently, both strengths of Mix 3 and Mix 4 increase with rates of 16.91% and 13.12% and 12.84% and 3.21%, respectively, when 0.1% and 0.2% fibre were added into concrete. This could be attributed to the fact that the fibre could restrict cracked samples to support higher loads than concrete without fibre. Afterwards, both strengths of samples of Mix 5 to Mix 7 fluctuate at around 3.2 MPa and 6.2 MPa, respectively. This can be interpreted as the fibre not being able to significantly improve the strength when it took up more than 0.3%. A possible explanation could be that excess gravel, which can bear higher loads, was substituted by the fibre. Additionally, the splitting tensile strength of concrete of Mix 4 to Mix 7 was higher than that of Mix 1. The concrete in Mix 7 had the highest splitting tensile strength and flexural strength of 3.224 MPa and 6.294 MPa, respectively.

### 4.2. Self-Sealing Capacity

#### 4.2.1. Crack Size Measurement

1. Visual Inspection

**Figure 10:**
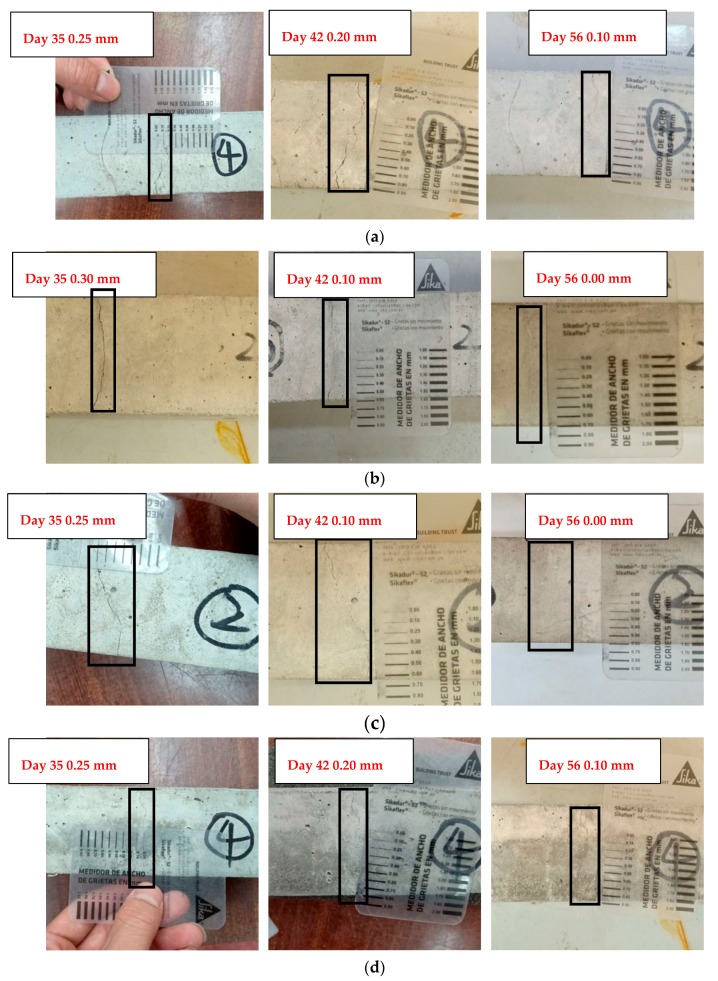

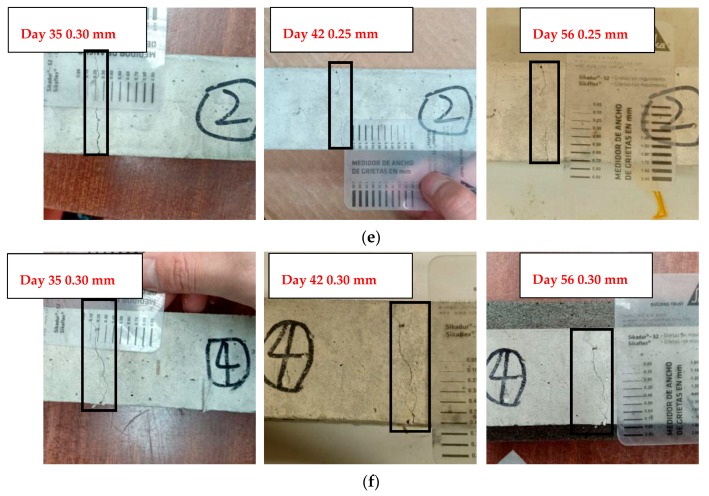
Crack sealing phenomenon of samples. (**a**) Changes in crack width of Mix 2 on days 35, 42, and 56; (**b**) changes in crack width of Mix 3 on days 35, 42, and 56; (**c**) changes in crack width of Mix 4 on days 35, 42, and 56; (**d**) changes in crack width of Mix 5 on days 35, 42, and 56; (**e**) changes in crack width of Mix 6 on days 35, 42, and 56; (**f**) changes in cracks width of Mix 7 on days 35, 42, and 56.

**Figure 11:**
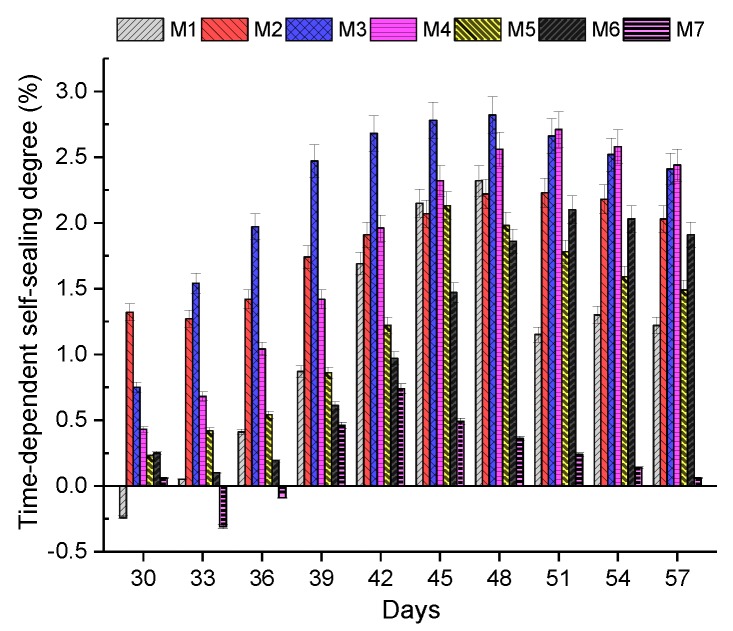
Time-dependent self-sealing degree.

**Figure 12:**
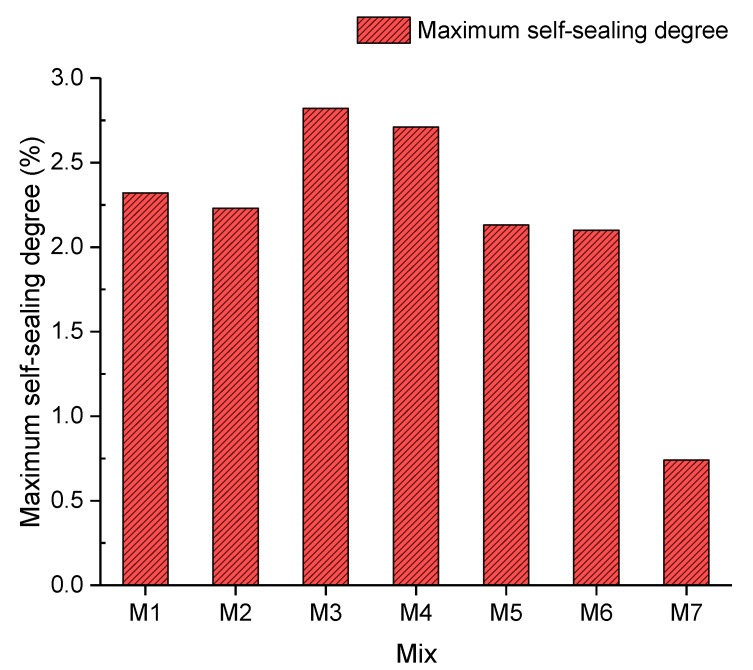
Maximum self-sealing degree.

Visual inspection cannot be the main method of measuring the self-sealing capacity because cracks do not propagate along lines. Moreover, there has been no research about the sealing directions of cracks. This means that it is not clear whether cracks are sealed from the bottom or the top [15]. Nevertheless, visual inspection could assist with observing the self-sealing phenomenon on the surface of cracks in concrete. Figure 10 shows pictures of sealed cracks in fibre-reinforced concrete from Mix 2 to Mix 7 on days 35, 42, and 56 after cracks’ appearance. For cracks in the concrete of Mix 2 and Mix 5, the widths changed from 0.25 mm to 0.10 mm between day 35 and day 42. Subsequently, both crack widths of Mix 2 and Mix 5 narrowed to 0.10 mm until day 56. Moreover, the widths of the cracks in the concrete of Mix 6 reduced from 0.30 mm to 0.25 mm between day 35 and day 42. They then held steady for the next 14 days. With regard to the widths of the cracks in the concrete of Mix 3 and Mix 4, they reduced from 0.30 mm and 0.25 mm, respectively, to 0.10 mm within the first seven days after sealing. Afterwards, the widths of both cracks reduced to 0.00 mm from day 42 to day 56. Considering the cracks in the concrete of Mix 7, the widths stayed constant at 0.30 mm between day 35 and day 56. All in all, for Mix 2, Mix 5, and Mix 6, cracks in concrete partially sealed after 56 days. Regarding Mix 7, no change was observed on the surface of cracks. With regards to Mix 3 and 4, cracks were completely sealed at the end of the sealing process. The concrete samples in Mix 3 and Mix 4 had a better self-sealing capacity than those in the rest of the mixes. Moreover, the concrete samples in Mix 2, Mix 5, and Mix 6 exhibited a lower self-sealing capacity than those in Mix 3 and Mix 4. Furthermore, the self-sealing phenomenon of samples in Mix 7 was negligible. This performance was in accordance with the maximum self-sealing degree in Figure 11 and Figure 12 obtained by the UPV method.

2. Damage Degree

The damage degree is utilised to define the initial crack size. The damage degree of concrete in each mix is listed in Table 7. As is shown, they are minimal from 3.3% to 6.9%, so differences in UPV results before and after cracking are relatively small. More specifically, the average damage degree is from 6.03% to 6.63% between Mix 1 and Mix 4, 4.77% and 4.27%, respectively, for Mix 5 and Mix 6, and 3.5% for Mix 7. However, the damage degree in the paper of Zhong and Yao is from 15% to 78% [26] and the damage degree is between 9.7% and 68.9% in the paper of Abd-Elmoaty [13]. Moreover, the crack width of concrete in each mix was between 0.25 mm and 0.30 mm, as demonstrated in Table 5. All in all, a tiny difference in the damage degree and the crack sizes of concrete between Mix 1 and Mix 7 was found in this article. Therefore, it can be concluded that the initial crack sizes of concrete in each mix are almost the same. It is crucial to employ samples with the same initial size of cracks in order to assess the influence of different dosages of the fibre on the self-sealing capacity. In this way, the self-sealing capacity of concrete samples with different proportions of the fibre can be measured accurately.

#### 4.2.2. Time-Dependent Self-Sealing Degree

Figure 11 shows the time-dependent self-sealing degree of concrete in the seven mixes. With regards to Mix 2, Mix 6, and Mix 7, the self-sealing degrees of them went through slight drops in the first five days after crack generation, which indicates the expansion of cracks. The self-sealing degree of Mix 7 decreased from 0.06 to −0.31% between the 30th and 33rd days. This means that the cracks in Mix 7 expanded in comparison with the original cracks. A possible reason for crack expansion could be that precipitation for sealing cracks could be easily seen in the early sealing stage because of the tension of water. Meanwhile, the self-sealing degree of Mix 2 and Mix 6 increased until the 51st day. Regarding Mix 7, the self-sealing degree increased from the 33rd to the 42nd day, and then dropped from 0.74% to 0.06% between day 42 and day 58. With regards to the rest of the mixes, the self-sealing degree of Mix 1, Mix 3, Mix 4, and Mix 5 increased from day 30 to day 48, by day 48, by day 51, and by day 45, respectively. Then the self-sealing degree of these mixes started dropping in the next few days. With regards to Figure 11, it can be seen that EFHDFRC in all mixes had the maximum self-sealing degree between day 42 and day 51 after casting concrete. This could be because the fibre can effectively bridge cracks at the early stage of sealing. However, the tension of fibres would be decreased and then the ability to combine cracked samples of fibres would be reduced because of long immersion in water.

#### 4.2.3. Maximum Self-Sealing Degree

In accordance with Figure 12, the maximum self-sealing degree, which indicates the highest percentage of sealed cracks of concrete, in each mix is revealed. According to Figure 12, the maximum self-sealing degree decreased from 2.32% to 2.23%, which represents a negligible drop from Mix 1 to Mix 2, by incorporating 5% crumb rubber. It could be interpreted that 5% crumb rubber had little effect on the maximum self-sealing degree of concrete, compared with plain concrete [43]. Compared with Mix 2 and Mix 3, the maximum self-sealing degree soared from 2.23% to 2.82% with a sealing efficacy increase of 20.92%. The maximum self-sealing degree of samples in Mix 3 was the highest, 2.82%. This means that the fibre could bridge concrete blocks to prevent further expansion and stimulate the self-sealing performance. Furthermore, the fibre could act as cores of precipitation, which could help to seal cracks by increasing the speed of precipitation formation. Subsequently, the maximum self-sealing degree plummeted from 2.82% to 0.74% between Mix 3 and Mix 7. A first possible explanation for this could be that excessive fibre lost the ability to bridge cracked samples as a result of a long soaking in the curing tank. Another possible reason could be that the cracks were filled up with the fibre and there was no space for forming deposits to seal cracks. The reason is that fibre has a higher density of distribution in cracks than gravel, and hence more chances of occupying the space for the rehydration of unreacted cement. Moreover, the binding force between the fibre and concrete was not as strong as that between the precipitation and concrete. Moreover, the ultrasound velocities passing through the fibre and deposition are different. Thus, the maximum self-sealing degree dropped with a growing proportion of the fibre.

#### 4.2.4. Regained Compressive Strength

**Figure 13:**
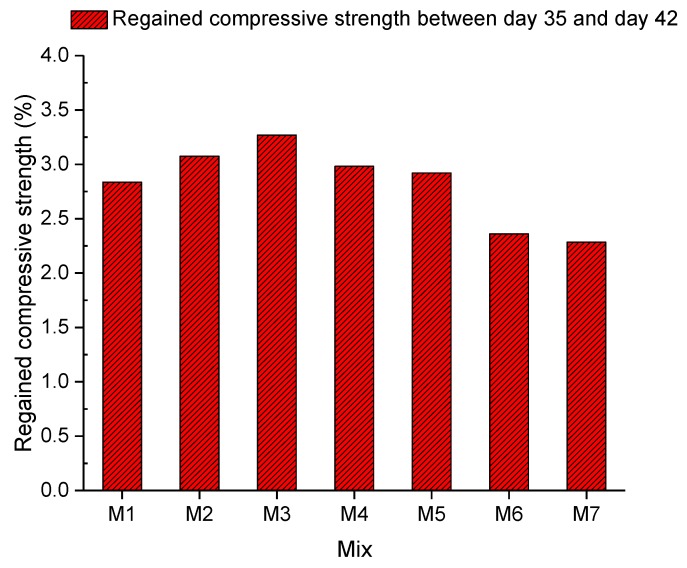
Regained compressive strength between day 35 and day 42.

In Figure 13, the self-sealing capacity of EFHDFRC obtained by measuring the regained compressive strength between days 35 and 42 after casting concrete is exhibited. The compressive strength regained between days 35 and 42 of seven concrete mixes is shown in Figure 13. The regained compressive strength increased from 2.836% to 3.075% between Mix 1 and Mix 2 with the additional 5% of crumb rubber from day 35 to day 42. Meanwhile, the maximum self-sealing degree of Mix 2 was lower than that of Mix 1. This could be because crumb rubber could expand to seal cracks at the early age of concrete to stimulate the self-sealing capacity of concrete. Nevertheless, crumb rubber could overexpand to the point of sealing failure and then the crumb rubber would start to shrink. Thus, the self-sealing capacity of concrete could drop with crumb rubber. Subsequently, the self-sealing capacity continuously rose to 3.269% in Mix 3. This could be attributed to the self-sealing capacity enhancement that resulted from adding the fibre. The fibre could bridge cracked concrete blocks to prevent overexpansion of cracks. Moreover, the unreacted cement remaining in concrete started to produce precipitation to seal cracks and to combine cracked concrete samples through the rehydration process. Afterwards, the self-sealing capacity decreased from 3.269% to 2.284% between Mix 3 and Mix 7. All in all, the regained compressive strength results shown in Figure 13 were from 2.284% to 3.269% between Mix 1 and Mix 7. With regard to previous papers on the regained compressive strength evaluation, 26.8% and 42.8% were found in the compressive strength recovery [5,26,44,45,46,47]. It is obvious that the regained compressive strengths in this article were significantly smaller than those in previous papers [48,49]. Thus, the regained compressive strength, ranging from 2.284% to 3.269% in this study, cannot be interpreted as a clear difference to be used in the assessment of the self-sealing capacity. The reason for the low regained compressive strength could be that the crack sizes mentioned in the crack size measurement in Section 4.2.1 were relatively small. If hydrated, reacted cement could produce precipitation for combining cracked samples that have an insufficient surface area due to the small crack size.

## 5. Conclusions

This study reveals the effect of fibre on the self-sealing capacity of EFHDFRC. The UPV result shows that concrete with 0.1% and 0.2% of the fibre had a considerable self-sealing capacity. The incorporation of 5% crumb rubber into concrete lowered the compressive strength, flexural strength, and splitting tensile strength by 30.66%, 29.20%, and 23.25%, respectively. Subsequently, different proportions of the fibre were applied to assess their influences on the mechanical properties of concrete with crumb rubber. With regards to the compressive strength among concretes with fibre, 0.1% fibre had the best performance, which was 53.70 MPa. Moreover, concrete with 0.4% of the fibre had the highest flexural and splitting tensile strength (6.294 MPa and 3.224 MPa, respectively). To evaluate the self-sealing capacity of concrete, the UPV method, the damage degree, the regained compressive strength, and visual inspection were employed. According to the time-dependent self-sealing degree in Figure 11, it was found that concrete in all mixes showed their maximum self-sealing degree between days 42 and 51 after casting concrete. Compared with the maximum self-sealing degree of mixes in Figure 12, concrete with 0.1% fibre had the best self-sealing degree, 2.82. Moreover, the self-sealing degree of concrete decreased with a growing proportion of the fibre, also leading to a smaller damage degree. The regained compressive strength results of concrete (2.284–3.269%) between days 35 and 42 after casting concrete, as shown in Figure 13, confirm that additional fibre can only seal cracks but hardly contributes to compressive strength recovery. Moreover, crumb rubber could enhance the self-sealing capacity at the early age of concrete by expansion. However, it could overexpand to lose the sealing ability. All in all, adding fibre to concrete can facilitate crack sealing. All told, 0.1% fibre confers the highest self-sealing capacity.

This work contributes to the research on how the self-sealing capacity of EFHDFRC is affected by the addition of fibre. To develop EFHDFRC with excellent self-sealing performance and strength recovery, water-swelling rubber is highly recommended due to its impressive expansion capacity. In order to decrease premature swelling, epoxy resin could be used because it is waterproof. Moreover, X-ray computed tomography (XCT) is suggested for analysing the distribution of water-swelling rubber. Furthermore, a sensor needs to be developed and installed on rubber to investigate the tensile strain for bridging cracked concrete. Furthermore, the damping ratio, affected by the addition of rubber, needs to be investigated further.

## Figures and Tables

**Table 1:** Properties of Duras S500 fibre.

Description	Properties
Fibre length	48 mm
Diameter	0.7 mm
Number of fibres per kg	58.714
Elastic Modulus	6000 MPa
Tensile strength	470 MPa
Density	0.922 kg/dm^3^
Melting point	165 °C
Ignition temperature	>360 °C

**Table 2:** Concrete design.

Mix	Ingredients	Cement	Water	Gravel	Sand	Crumb Rubber (CR)	Fibre (F)
M1	Reference	530	233	986	630	–	–
M2	5% CR	530	233	986	598.5	31.5	–
M3	5% CR, 0.1% F	530	233	983.621	598.5	31.5	2.379
M4	5% CR, 0.2% F	530	233	981.242	598.5	31.5	4.758
M5	5% CR, 0.3% F	530	233	978.863	598.5	31.5	7.137
M6	5% CR, 0.35% F	530	233	977.673	598.5	31.5	8.327
M7	5% CR, 0.4% F	530	233	976.484	598.5	31.5	9.516
Unit: kg							

**Table 3:** Gradation of aggregates.

No.	Sieves (mm)	Weight Retained (g)	Retained (%)	Cumulative Retained (%)	Finer (%)
1	20	0	0	0	100
2	16	0	0	0	100
3	10	855	21.5	21.5	78.5
4	6.7	2730	68	89.5	10.5
5	4.75	335	8.5	98	2
6	Base	80	2	100	0
	Total	4000			

**Table 4:** Testing details.

Testing	Specifications	Specimen Dimensions (mm)	Number of Samples
Compressive strength	BS EN 12390-3	100 × 100 × 100	9
Splitting tensile strength	BS EN 12390-6	Ø100 × L 200	3
Flexural strength	BS EN 12390-5	100 × 100 × 500	3
Time-dependent self-sealing degree	BS EN 12504-4	50 × 50 × 250	4
Regained compressive strength	BS EN 12390-3	100 × 100 × 100	3
Damage degree	–	50 × 50 × 250	3
Visual inspection	–	50 × 50 × 250	3

**Table 5:** Initial crack widths of concrete in each mix.

Mix No.	Initial Crack Width (mm)
M1	0.25
M2	0.25
M3	0.30
M4	0.25
M5	0.25
M6	0.30
M7	0.30

**Table 6:** Regained compressive strength test instruction.

Day 28 (Cracks Generation)	Day 35	Day 42
3 reference samples	3 reference samples	3 reference samples
Sample A, B, C (1st test)	Sample A, B, C (2nd test)	–
Samples D, E, F (1st test)	–	Samples D, E, F (2nd test)

**Table 7:** UPV values to define damage degree.

Mix No.	Specimen No.	UPV before Cracking (km/s)	UPV after Cracking (km/s)	Damage Degree (%)	Average Damage Degree (%)	S.D.
M1	1	4.513	4.248	5.9	6.03	0.115
	2	4.546	4.277	6.1		
	3	4.519	4.246	6.1		
M2	1	4.129	3.873	6.2	6.63	0.379
	2	4.098	3.818	6.8		
	3	4.115	3.831	6.9		
M3	1	4.139	3.874	6.4	6.27	0.153
	2	4.184	3.929	6.1		
	3	4.125	3.865	6.3		
M4	1	4.214	3.953	6.2	6.03	0.208
	2	4.151	3.914	5.8		
	3	4.122	3.870	6.1		
M5	1	4.201	3.995	4.9	4.77	0.153
	2	4.151	3.951	4.8		
	3	4.167	3.975	4.6		
M6	1	4.112	3.928	4.4	4.27	0.153
	2	4.114	3.937	4.3		
	3	4.122	3.951	4.1		
M7	1	4.181	4.023	3.8	3.50	0.265
	2	4.182	4.044	3.3		
	3	4.196	4.055	3.4		
